# Inhibition of ROS-Activated p38MAPK Pathway is Involved in the Protective Effect of H_2_S Against Chemical Hypoxia-Induced Inflammation in PC12 Cells

**DOI:** 10.1007/s11064-013-1044-x

**Published:** 2013-04-27

**Authors:** Aiping Lan, Wenming Xu, Hui Zhang, Xiaoxiao Hua, Dongdan Zheng, Runmin Guo, Ning Shen, Fen Hu, Jianqiang Feng, Donghong Liu

**Affiliations:** 1 Department of Physiology, Zhongshan School of Medicine, Sun Yat-sen University, No. 74, The Second Zhongshan Road, Guangzhou, 510080 Guangdong People’s Republic of China; 2 Department of Internal Medicine, Region of Huangpu, The First Affiliated Hospital, Sun Yat-sen University, Guangzhou, 510700 Guangdong People’s Republic of China; 3 Hospital of Orthopedics, General Hospital of Guangzhou Military Command of PLA, Guangzhou, 510010 Guangdong People’s Republic of China; 4 Department of Anesthesiology, The First Affiliated Hospital, Sun Yat-sen University, Guangzhou, 510700 Guangdong People’s Republic of China; 5 Department of Cardiology, Region of Huangpu, The First Affiliated Hospital, Sun Yat-sen University, Guangzhou, 510700 Guangdong People’s Republic of China; 6 Division of Ultrasound, The First Affiliated Hospital, Sun Yat-sen University, No. 58, The Second Zhongshan Road, Guangzhou, 510080 Guangdong People’s Republic of China

**Keywords:** Hydrogen sulfide, Hypoxia, Inflammation, p38 MAPK, Nitric oxide, Cobalt chloride

## Abstract

We have demonstrated the neuroprotection of hydrogen sulfide (H_2_S) against chemical hypoxia-induced injury by inhibiting p38MAPK pathway. The present study attempts to evaluate the effect of H_2_S on chemical hypoxia-induced inflammation responses and its mechanisms in PC12 cells. We found that treatment of PC12 cells with cobalt chloride (CoCl_2_, a hypoxia mimetic agent) enhanced IL-6 secretion, nitric oxide (NO) generation and expression levels of inducible nitric oxide synthase (iNOS) and neuronal nitric oxide synthase (nNOS). L-canavanine, a selective iNOS inhibitor, partly blocked CoCl_2_-induced cytotoxicity, apoptosis and mitochondrial insult. In addition, 7-Nitroindazole (7-NI), an inhibitor of nNOS, also partly attenuated the CoCl_2_-induced cytotoxicity. The inhibition of p38MAPK by SB203580 (a selective p38MAPK inhibitor) or genetic silencing of p38MAPK by RNAi (Si-p38) depressed not only CoCl_2_-induced iNOS expression, NO production, but also IL-6 secretion. In addition, N-acetyl-l-cysteine, a reactive oxygen species (ROS) scavenger, conferred a similar protective effect of SB203580 or Si-p38 against CoCl_2_-induced inflammatory responses. Importantly, pretreatment of PC12 cells with exogenous application of sodium hydrosulfide (a H_2_S donor, 400 μmol/l) for 30 min before exposure to CoCl_2_ markedly attenuated chemical hypoxia-stimulated iNOS and nNOS expression, NO generation and IL-6 secretion as well as p38MAPK phosphorylation in PC12 cells. Taken together, we demonstrated that p38MAPK-iNOS pathway contributes to chemical hypoxia-induced inflammation and that H_2_S produces an anti-inflammatory effect in chemical hypoxia-stimulated PC12 cells, which may be partly due to inhibition of ROS-activated p38MAPK-iNOS pathway.

## Introduction

Hydrogen sulfide (H_2_S) has been considered for decades only a cytotoxic gas. However, H_2_S, recently recognized as the third “gas signal molecule” alongside nitric oxide (NO) and carbon monoxide (CO) [1], has been attracted extensive attention due to its multiple physiological and pathophysiological effects in various body systems [1–5]. Accumulating evidence suggests that H_2_S may serve as an important neuroprotective agent. One of the most important mechanisms underlying H_2_S protection is its antioxidation. H_2_S exerts its protective effect not only by increasing reduced glutathione (GSH) in neurons [6], but also by directly scavenging superoxide anions, hydrogen peroxide (H_2_O_2_) [7] and peroxynitrite [8] to suppress oxidative stress. However, the exact role of H_2_S in inflammation is controversial since both pro- and anti-inflammatory effects have been revealed [9]. It has been found that H_2_S exhibits pro-inflammatory role in animal models of pancreatitis, septic/endotoxic, and hemorrhagic shock [4, 10–12]. However, in the lipopolysaccharide-stimulated microglia and astrocytes, H_2_S can protect against inflammatory effect [13]. Additionally, we have also demonstrated H_2_S has an anti-inflammatory effect in the chemical hypoxia-stimulated skin cells [14], but whether H_2_S prevents from the chemical hypoxia-induced inflammation in PC12 cells has not been reported.

Cobalt chloride (CoCl_2_) is a well-known mimetic agent of hypoxia/ischemia, which induces oxidative stress [15–17]. PC12 cells have been widely used as a well-established model for exploring many aspects of the cellular biology of neurons. Hence, in this study, PC12 cells were treated with CoCl_2_ to establish the chemical hypoxia-induced injury model, in which we investigated: (a) the effect of H_2_S on the pro-inflammatory factors induced by CoCl_2_; (b) role of p38 mitogen-activated protein kinase (MAPK) in the CoCl_2_-induced inflammation; (c) role of inhibition of p38MAPK- iNOS pathway in the anti-inflammatory effect of H_2_S. We found that CoCl_2_ induces not only PC12 cells injury and inflammatory responses (including increases in the expression of iNOS, production of NO and secretion of IL-6), but also the activation of p38MAPK pathway which contributes to the CoCl_2_-induced injury and inflammation and that exogenous H_2_S protects PC12 cells against the CoCl_2_-induced inflammation by inhibition of p38MAPK-iNOS pathway activated by ROS.

## Materials and Methods

### Materials

Sodium hydrosulfide (NaHS), CoCl_2_, N-acetyl-l-cysteine (NAC), L-canavanine, 7-Nitronidazole (7-NI), 5,5′,6,6′-tetrachloro-1,1′,3,3′-tetraethylbenzimidazol-carbocanine iodide (JC-1), Hoechst 33258 and propidium iodide (PI) were purchased from Sigma-Aldrich (St Louis, MO, USA). The cell counter kit-8 (CCK-8) was purchased from Dojindo Lab (Kumamoto, Japan). DMEM medium and fetal bovine serum (FBS) were supplied by Gibco BRL (Grand Island, NY, USA). Anti-p38 antibody, anti-p-p38 antibody and SB203580 were purchased from Cell Signaling Technology (Boston, MA, USA). Specific monoclonal anti-iNOS or anti-nNOS antibody was obtained from Santa cruz Biotechnology, Inc (Delaware Avenue, CA, USA). The Griess reagent assay kit and L-canavanine were obtained from Beyotime Institute of Biotechnology (Haimen, China). Enzyme-linked immunosorbent assay (ELISA) kit was provided by Boster Bio-Engineering Limited Company (Wuhan, China). Anti-β-actin antibody, horseradish peroxidase (HRP)-conjugated secondary antibody and BCA protein assay kit were purchased from KangChen Bio-tech, Inc (Shanghai, China). Enhanced chemiluminescence (ECL) solution was purchased from KeyGen Biotech (Nanjing, China).

### Cell Culture and Treatments

The differentiated PC12 cells, a rat pheochromocytoma cell line, were purchased from Sun Yat-sen University Experimental Animal Center (Guangzhou, China). The cells were grown in DMEM medium supplemented with 10 % FBS at 37 °C under an atmosphere of 5 % CO_2_ and 95 % air. According to our previous study [18], chemical hypoxia was achieved by adding CoCl_2_ at 600 μmol/l into the medium and the cells were incubated in the presence of CoCl_2_ for the indicated times. The anti-inflammatory effects of H_2_S were observed by administering 400 μmol/l NaHS (a donor of H_2_S) for 30 min prior to exposure to CoCl_2_ for 24 h. NAC or SB203580 (a selective inhibitor of p38MAPK) was administered 60 min prior to exposure to 600 μmol/l CoCl_2_ for 24 h. L-canavanine was administered 60 min prior to exposure to 600 μmol/l CoCl_2_ for 24 h.

### Cell Viability Assay

PC12 cells in logarithmic growth curves were inoculated onto 96-well plates at a concentration of 1 × 10^4^/ml, and the cell viability was assessed by the CCK-8 assay. After the indicated treatments, 10 μl CCK-8 solution was added to each well of the plate and the cells in the plate were incubated for 4 h in the incubator. The absorbance at 450 nm was measured with a microplate reader (Molecular Devices, Sunnyvale, CA, USA). Means of 4 wells optical density (OD) in the indicated groups were used to calculate percentage of cell viability according to the formula below: cell viability (%) = (OD_treatment group_/OD_control group_) × 100 %. The experiment was repeated 3 times.

### Nuclear Staining for Assessment of Apoptosis

Apoptotic cell death was determined by the Hoechst33258 staining method. Cells were plated in 35 mm dishes at a density of 1 × 10^6 ^cells/well. Cells were preconditioned with 400 μmol/l NaHS for 30 min, subsequently exposed to 600 μmol/l CoCl_2_ for 48 h. To test the role of iNOS/NO in the CoCl_2_-induced apoptosis, cells were pretreated with the iNOS inhibitor L-canavanine for 60 min prior to exposure of cells to CoCl_2_ for 48 h. At the end of the indicated treatments, cells were harvested and fixed with 4 % paraformaldehyde in 0.1 mol/l phosphate–buffered saline (PBS, pH 7.4) for 10 min. After rinsing with PBS, the nuclear DNA was stained with 5 mg/ml Hoechst33258 dye for 10 min before being rinsed briefly with PBS and then visualized under a fluorescence microscope (Bx50-FLA; Olympus, Tokyo, Japan). Viable cells displayed a uniform blue fluorescence throughout the nucleus, whereas apoptotic cells showed condensed and fragmented nuclei.

### Flow Cytometry Analysis of Apoptosis

PC12 cells were treated as previously described. Adherent cells were enzymatically digested with trypsin (2.5 mg/ml). Following centrifugation at 350*g* for 10 min, the supernatant was removed. Cells were washed twice with PBS and fixed with 70 % ice-cold ethanol. Cells were then centrifuged at 350*g* for 10 min, washed twice with PBS and adjusted to a concentration of 1 × 10^6 ^cells/ml. Then, 0.5 ml RNase (1 mg/ml in PBS) was added to a 0.5 ml cell sample. After gentle mixing with PI (at a terminal concentration of 50 mg/l), mixed cells were filtered and incubated in the dark at 4 °C for 30 min before flow cytometric analysis. The PI fluorescence of individual nuclei was measured by a flow cytometer (FCM) (Beckman-Coulter, Los Angeles, CA, USA). (excitation: 488 nm, emission: 615 nm). The research software matched with FCM was used to analyze all the data of DNA labeling. In the DNA histogram, the amplitude of the sub-G1 DNA peak, which is lower than the G1 DNA peak, represents the number of apoptotic cells. The experiment was repeated 3 times.

### Measurement of Mitochondrial Membrane Potential

To determine the mitochondrial membrane potential (MMP), the lipophilic cationic probe JC-1 was used. In living cells, JC-1 exists either as a green fluorescent monomer at low membrane potential or as an orange-red fluorescent J-aggregate at high membrane potentials. The ratio of red/green JC-1 fluorescence is dependent on the MMP. In the present study, PC12 cells were cultured in 24 well plates and suffered from the indicated treatments. JC-1 (5 mg/l) was added and incubated for 30 min at 37 °C and the fluorescence was observed over the entire field of vision by a inverted fluorescence microscope (Axio Observer Z1, Carl Zeiss, Germany) connected to an imaging system. The ratio of red/green fluorescent density from 4 random fields was analyzed by AxioVision Microscope Software of Carl Zeiss. The experiment was repeated 5 times.

### NO Determination in Culture Supernatant

Accumulated nitrite, an indicator of the production of NO, was measured after the treatment with 600 μmol/l CoCl_2_ for 48 h in PC12 cells. Nitrite was measured in the culture supernatant using a commercial kit. Briefly, 50 μl aliquots of cell culture medium from each dish were collected and mixed with 100 μl of Griess reagent (50 μl of 1 % sulfanilamide + 50 μl of 0.1 % naphthylethylenediamine dihydrochloride in 2.5 % H_3_PD_4_) in a 96-well microtiter plate. The absorbance of NO_2_^−^ was read at 520 nm using a plate reader. In the preliminary experiments, NaHS (400 μmol/l) was found to have no significant effect on the Griess reaction (data not shown).

### ELISA for the Detection of IL-6 in Culture Supernatant

Secretion of IL-6 was determined by ELISA. PC12 cells were plated in 96-well plates. After the cells were treated as indicated, the relative content of secreted inflammatory factor in the supernatant was measured by ELISA according to the manufacturer’s instruction. The relative content of the inflammatory factor in culture medium was normalized to cell viability measured by CCK-8 assay. The experiment was carried out in triplicate.

### Western Blot Assay for Expressions of Protein

After subjected to the indicated treatments, cells were harvested and lysed with cell lysis solution. Total proteins in the cell lysate were quantified using the BCA protein assay kit. Loading buffer was added to cytosolic extracts, and after boiling for 5 min, equal amounts of supernatant from each sample were fractionated by 10 % sodium dodecyl sulphate–polyacrylamide gel electrophoresis (SDS-PAGE). The total proteins in the gel were transferred into polyvinylidene difluoride (PVDF) membranes. The membranes were blocked for 1.5 h at room temperature in fresh blocking buffer (0.1 % Tween20 in Tris–buffered saline (TBS-T) containing 5 % fat-free milk) and then incubated with either anti-p38 (1:1,000 dilution), anti-p-p38 (1:1,000 dilution), anti-iNOS (1:500 dilution), anti-nNOS (1:1,000 dilution) or anti-β-actin antibody (1:5,000 dilution) in freshly prepared TBS-T with 3 % free-fat milk overnight with gentle agitation at 4 °C. Following three washes with TBS-T, membranes were incubated with HRP-conjugated goat anti-rabbit secondary antibody (1:3,000 dilution; Kangchen Biotech, shanghai, China) in TBS-T with 3 % fat-free milk for 1.5 h at room temperature. Membranes were washed three times with TBS-T, developed in ECL solution and visualized with X-ray film. Each experiment was repeated at least three times. For quantification, the films were scanned and analyzed by ImageJ 1.47i software.

### Gene Knockdown

Small interfering RNA (Si-RNA) against rat p38MAPK subunit mRNA (NM-031020) was synthesized by GenePharma Co., Ltd (Shanghai, China). The Si-RNA of p38 (Si-p38) and random non-coding RNA (Si-NC) were transfected, respectively, into PC12 cells using Lipofectamine 2000, according to the manufacturer’s instruction (Invitrogen, USA). Si-p38MAPK and Si-NC (50 nmol/l) were incubated with the cells for 6 h in order to transfect into the cells. Efficiency of genetic silencing by Si-RNA was evaluated by Western blot assay.

### Statistical Analysis

All data are expressed as the mean ± SEM. Differences between groups were analyzed by one-way analysis of variance (ANOVA) using SPSS 13.0 software, and followed by LSD post hoc comparison test. Statistical significance was defined as *P* < 0.05.

## Results

### CoCl_2_ Enhances iNOS Expression and NO Production in PC12 Cells

**Fig. 1:**
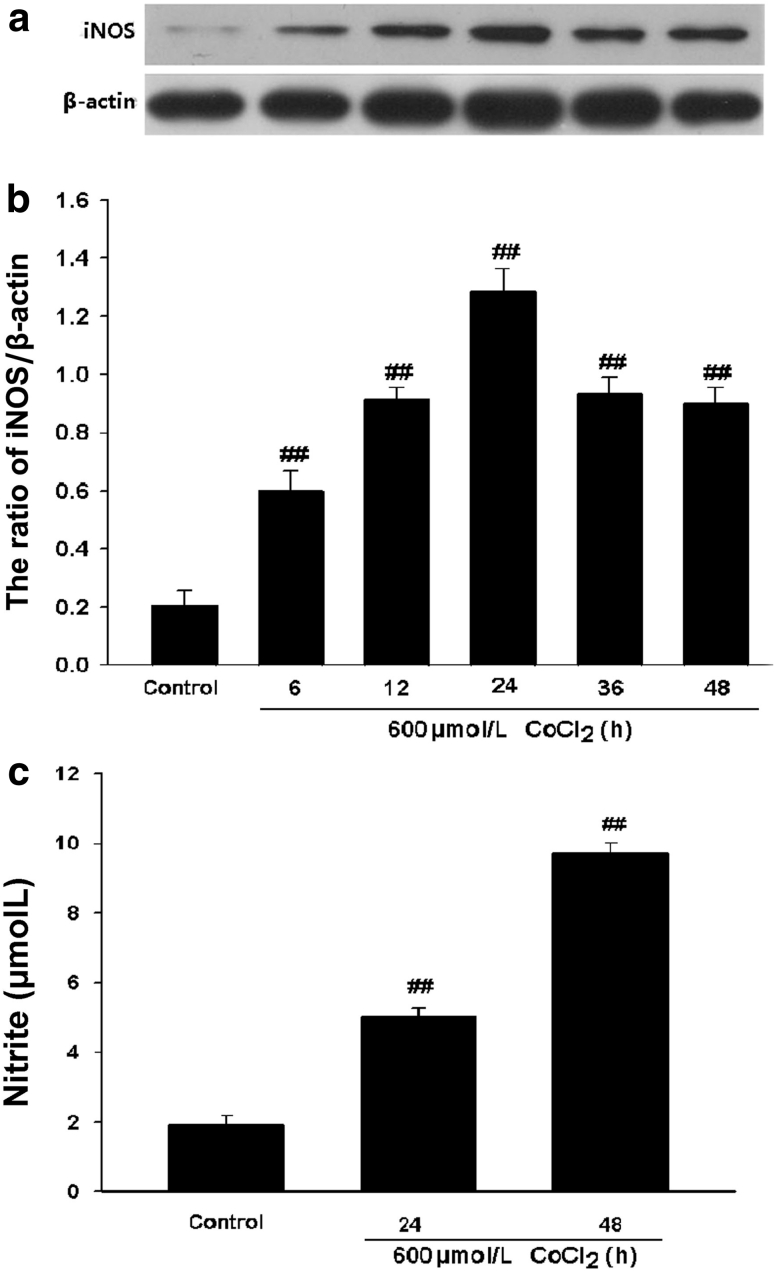
CoCl_2_ increases expression of iNOS and production of NO in PC12 cells. **a** PC12 cells were treated with 600 μmol/l CoCl_2_ for indicated times. The expression of iNOS was tested by Western blot assay. **b** Densitometric analysis of the results from (**a**). **c** PC12 cells were treated with 600 μmol/l CoCl_2_ for 24 or 48 h. Nitrite in the culture supernatant was determined using the Griess reagent as described in “Materials and Methods”. Data were presented as mean ± SEM (n = 3). ^##^ *P* < 0.01 versus control group

To explore the effect of CoCl_2_ on expression of iNOS and production of NO, PC12 cells were treated with 600 μmol/l CoCl_2_ for the indicated times ranging from 0 to 48 h. The findings of Western blot analysis showed that a significant increase in the expression of iNOS was firstly observed at 6 h, and reached a peak at 24 h, indicating that CoCl_2_ exposure evokes iNOS expression (Fig. 1a, b). In addition, as shown in Fig. 1c, the exposure of PC12 cells to 600 μmol/l CoCl_2_ also induced the overproduction of NO at specific times (i.e. 24 h, 48 h).

### NOS/NO Pathway Mediates CoCl_2_-Induced Injuries in PC12 Cells

**Fig. 2:**
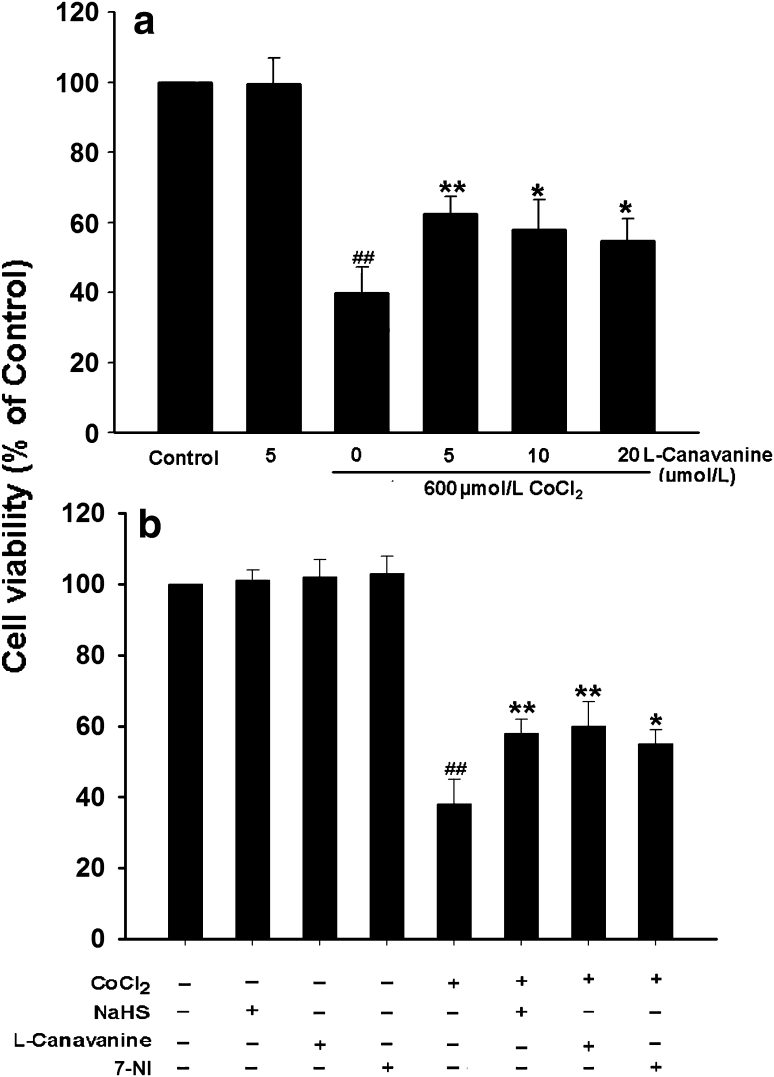
NaHS, L-canavanine or 7-nitroindazole inhibits CoCl_2_-induced cytotoxicity in PC12 cells. **a** PC12 cells were pretreated with L-canavanine, an inhibitor of iNOS, at indicated concentrations for 60 min before exposure to 600 μmol/l CoCl_2_ for 24 h. **b** PC12 cells were pretreated with 400 μmol/l NaHS for 30 min or 5 μmol/l L-canavanine for 60 min or 250 μmol/l 7-nitroindazole (7-NI), an inhibitor of nNOS, for 30 min prior to exposure to 600 μmol/l CoCl_2_ for 24 h. Cell viability was measured by the CCK-8 assay. Data were presented as mean ± SEM (n = 3). ^##^ *P* < 0.01 versus control group; *P < 0.05, **P < 0.01 versus CoCl_2_ group

To dissect the role of iNOS/NO pathway in the CoCl_2_-induced injuries. We firstly tested the effect of L-canavanine (a selective iNOS inhibitor) on the CoCl_2_-induced cytotoxicity in PC12 cells. As shown in Fig. 2a, b, exposure of PC12 cells to 600 μmol/l CoCl_2_ for 24 h obviously induced cytotoxicity, the cell viability was markedly reduced to (41.3 ± 4.4)% (*P* < 0.01). However, pretreatment of cells with 5 μmol/l L-canavanine for 60 min prior to CoCl_2_ exposure dramatically inhibited the CoCl_2_-induced cytotoxicity. The cell viability was increased to (62.1 ± 2.3) % (*P* < 0.01). L-canavanine at 5 μmol/l alone did not induce significant cytotoxicity. These findings indicated that iNOS/NO pathway mediates cytotoxicity induced by CoCl_2_ in PC12 cells. Similarly, pretreatment with 250 μmol/l 7-NI (an inhibitor of nNOS) for 30 min before CoCl_2_ exposure also obviously blocked the CoCl_2_-induced cytotoxicity (Fig. 2b). Furthermore, pretreatment of cells with 400 μmol/l NaHS for 30 min prior to CoCl_2_ exposure also inhibited the CoCl_2_-induced cytotoxicity (Fig. 2b).

**Fig. 3:**
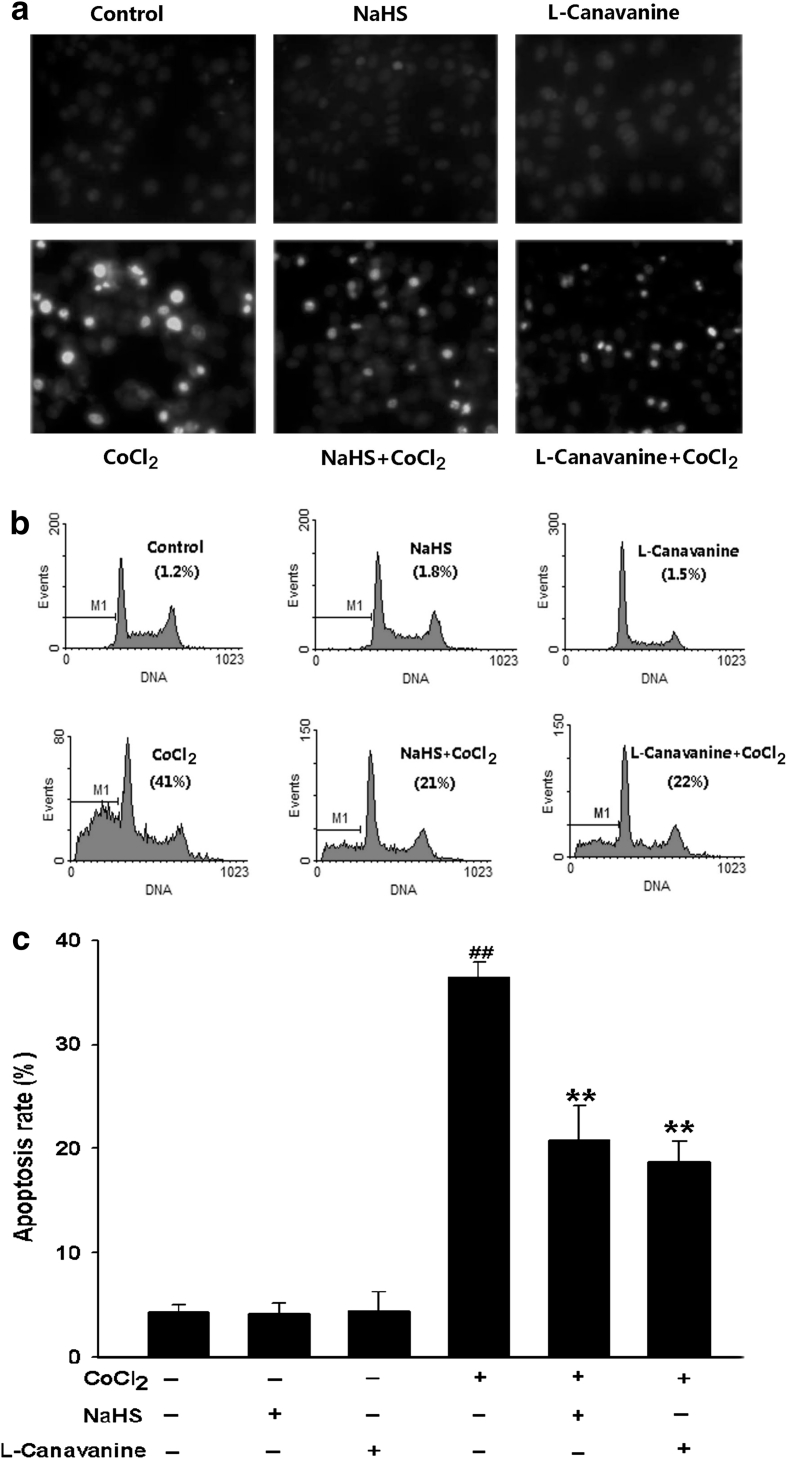
NaHS and L-canavanine block CoCl_2_-induced apoptosis in PC12 cells. **a** Morphological changes in apoptotic cells were assessed by Hoechst33258 staining. **b** Apoptosis rate was determined by FCM as described in “Materials and Methods”. Control group: Normal PC12 cells; NaHS group: cells were treated with 400 μmol/l NaHS for 30 min alone; L-canavanine group: cells were exposed to 5 μmol/l L-canavanine for 60 min alone; CoCl_2_ group: cells were treated with 600 μmol/l CoCl_2_ for 48 h; NaHS + CoCl_2_: cells were pretreated with 400 μmol/l NaHS for 30 min followed by 600 μmol/l CoCl_2_ treatment for 48 h. L-canavanine + CoCl_2_: cell were pretreated with 5 μmol/l L-canavanine for 60 min followed by 600 μmol/l CoCl_2_ treatment for 48 h. Data were presented as mean ± SEM (n = 5). ^##^ *P* < 0.01 versus control group; **P < 0.01 versus CoCl_2_ group

We secondarily examined the effect of L-canavanine on the CoCl_2_-induced apoptosis in PC12 cells. As shown in Fig. 3a, PC12 cells treated with 600 μmol/l CoCl_2_ for 48 h exhibited typical characteristics of apoptosis, including the condensation of chromatin, shrinkage of nuclear and a few of apoptotic bodies. However, pretreatment of cells with 5 μmol/l L-canavanine for 60 min before exposure to CoCl_2_ considerably decreased the CoCl_2_-induced apoptotic cells with nuclear condensation and fragmentation (Fig. 3a), L-canavanine alone did not markedly alter morphology or apoptotic percentage of PC12 cells. Moreover, the data from FCM analysis further demonstrated that exposure of cells to 600 μmol/l CoCl_2_ for 48 h increased the rate of apoptotic PC12 cells (Fig. 3b), which was markedly suppressed by pretreatment of cells with L-canavanine for 60 min (Fig. 3c). The data revealed that iNOS/NO pathway is involved in the CoCl_2_-induced apoptosis in PC12 cells. Additionally, pretreatment of cells with 400 μmol/l NaHS for 30 min before exposure CoCl_2_ also markedly inhibited the CoCl_2_-induced cell apoptosis (Fig. 3).

**Fig. 4:**
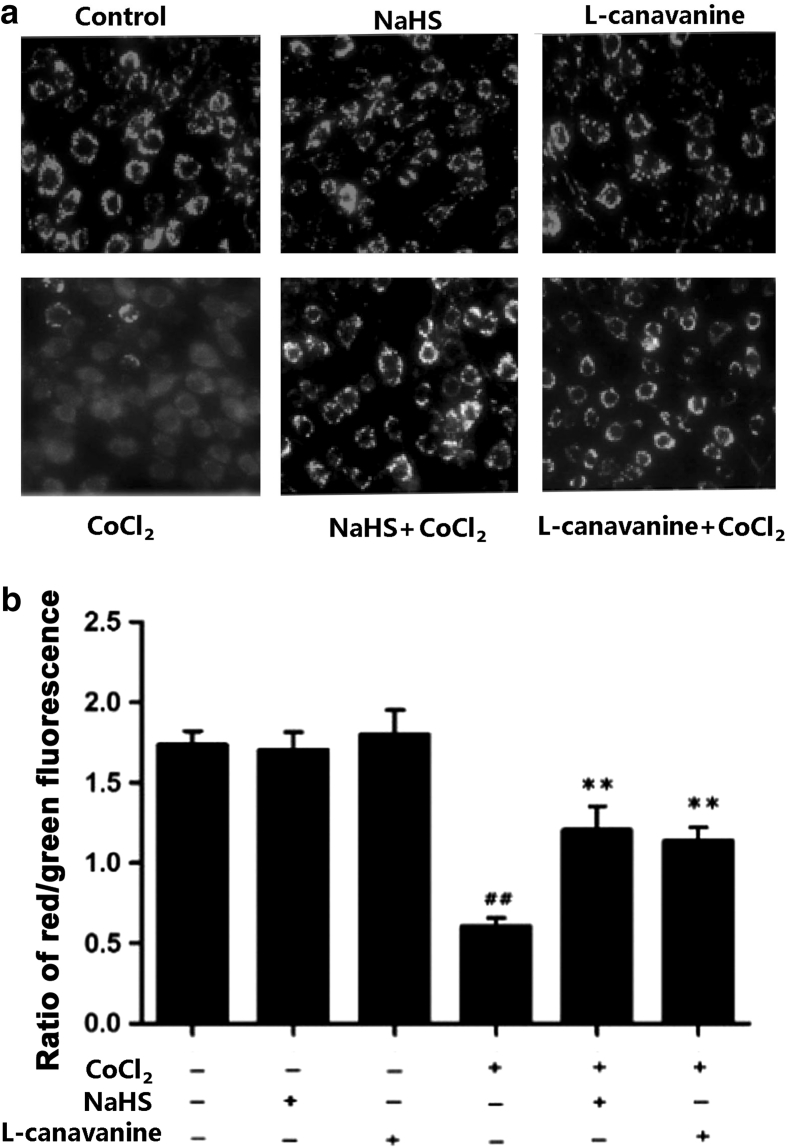
NaHS and L-canavanine attenuate CoCl_2_-induced mitochondrial insult. MMP was measured by JC-1 staining followed by photofluorography. Dual emission images (527 and 590 nm) represent the signals from monomeric (*green*) and J-aggregate (*red*) JC-1 fluorescence in PC12 cells. **a** Random micrographs of fluorescence in PC12 cells. Control group: normal PC12 cells; NaHS group: cells were exposed to 400 μmol/l NaHS for 30 min alone; L-canavanine group: cells were incubated with 5 μmol/l L-canavanine for 60 min alone; CoCl_2_ group: cells were treated with 600 μmol/l CoCl_2_ for 24 h; NaHS + CoCl_2_: cells were pretreated with 400 μmol/l NaHS for 30 min followed by exposure to 600 μmol/l CoCl_2_ for 24 h. L-canavanine + CoCl_2_: cells were pretreated with 5 μmol/l L-canavanine for 60 min followed by exposure to 600 μmol/l CoCl_2_ for 24 h. **b** Quantitative analysis of the ratio of *red*/*green fluorescence* in the indicated groups. Data were presented as mean ± SEM (n = 5). ^##^ *P* < 0.01 versus control group; **P < 0.01 versus CoCl_2_ group (Color figure online)

We thirdly determined the effect of L-canavanine on the CoCl_2_-induced mitochondrial insult. As shown in Fig. 4a and b, PC12 cells treated with 600 μmol/l CoCl_2_ for 24 h exhibited significant mitochondrial damage, evidenced by MMP loss as shown by a decrease in the ratio of red/green fluorescence. However, preconditioning with 5 μmol/l L-canavanine for 60 min prior to the CoCl_2_ treatment for 24 h markedly suppressed the CoCl_2_-induced dissipation of MMP, compared with CoCl_2_-treated cells (*P* < 0.01) (Fig. 4b). L-canavanine alone did not affect the MMP. These results suggested that iNOS/NO pathway is associated with the CoCl_2_-induced mitochondrial insult in PC12 cells.

### p38MAPK Activation Contributes to CoCl_2_-Induced iNOS Expression and NO Production in PC12 Cells

**Fig. 5:**
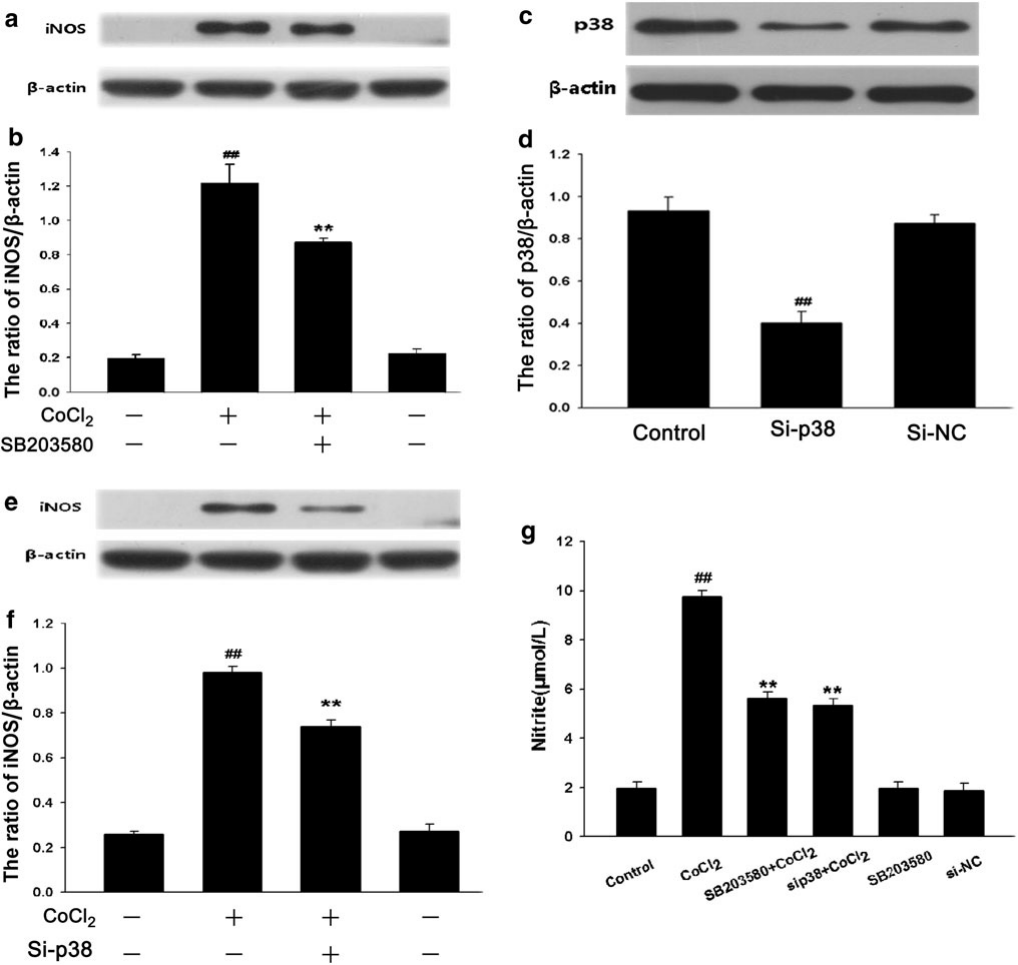
p38MAPK activation participates in CoCl_2_-induced iNOS expression and NO production in PC12 cells. The expression of iNOS was tested by Western blot assay. **a** PC12 cells were preconditioned with 20 μmol/l SB203580 for 60 min before exposure to 600 μmol/l CoCl_2_ for 24 h. **c** PC12 cells were co-cultured with small interfering RNA (Si-p38) or random non-coding RNA(Si-NC) for 6 h. **e** PC12 cells were co-incubated with Si-p38 for 6 h followed by treated with 600 μmol/l CoCl_2_ for 24 h. **b**, **d**, and **f** densitometric analysis of the results from **a**, **c**, and **e**, respectively. **g** PC12 cells were treated with 600 μmol/l CoCl_2_ for 48 h in the presence or absence of pretreatment with 20 μmol/l SB203580 for 60 min or co-cultured with Si-p38 for 6 h. Nitrite in the culture supernatant was determined using the Griess reagent as described in “Materials and Methods”. Data were presented as mean ± SEM (n = 5). ^##^ *P* < 0.01 versus Control group; **P < 0.01 versus CoCl_2_ group

To investigate whether activation of p38MAPK was involved in the CoCl_2_-induced increases in iNOS expression and NO production in PC12 cells. We examined the effect of inhibition of p38MAPK on the level of iNOS expression and NO production induced by CoCl_2_. The data of Western blot analysis showed that treatment of cells with CoCl_2_ for 24 h markedly increased the iNOS protein expression by 6-fold, compared with the control group (*P* < 0.01). However, pretreatment with 20 μmol/l SB203580, a selective inhibitor of p38MAPK, for 60 min before CoCl_2_ treatment, blocked the CoCl_2_-induced stimulatory effect on iNOS expression (Fig. 5a, b). Additionally, after p38MAPK was suppressed by small interfering RNA against p38 (Si-p38) (Fig. 5c, d), CoCl_2_-induced increase in iNOS expression was also markedly reduced (Fig. 5e, f). Furthermore, the pretreatment with SB203580 at 20 μmol/l for 60 min or co-incubation of cells with Si-p38 for 6 h markedly inhibited CoCl_2_-induced NO production (Fig. 5g). The above findings suggested that the activation of p38MAPK mediates, at least partly, the CoCl_2_-induced increases in iNOS expression and NO production.

### ROS Participate in CoCl_2_-Induced Activation of iNOS/NO System

**Fig. 6:**
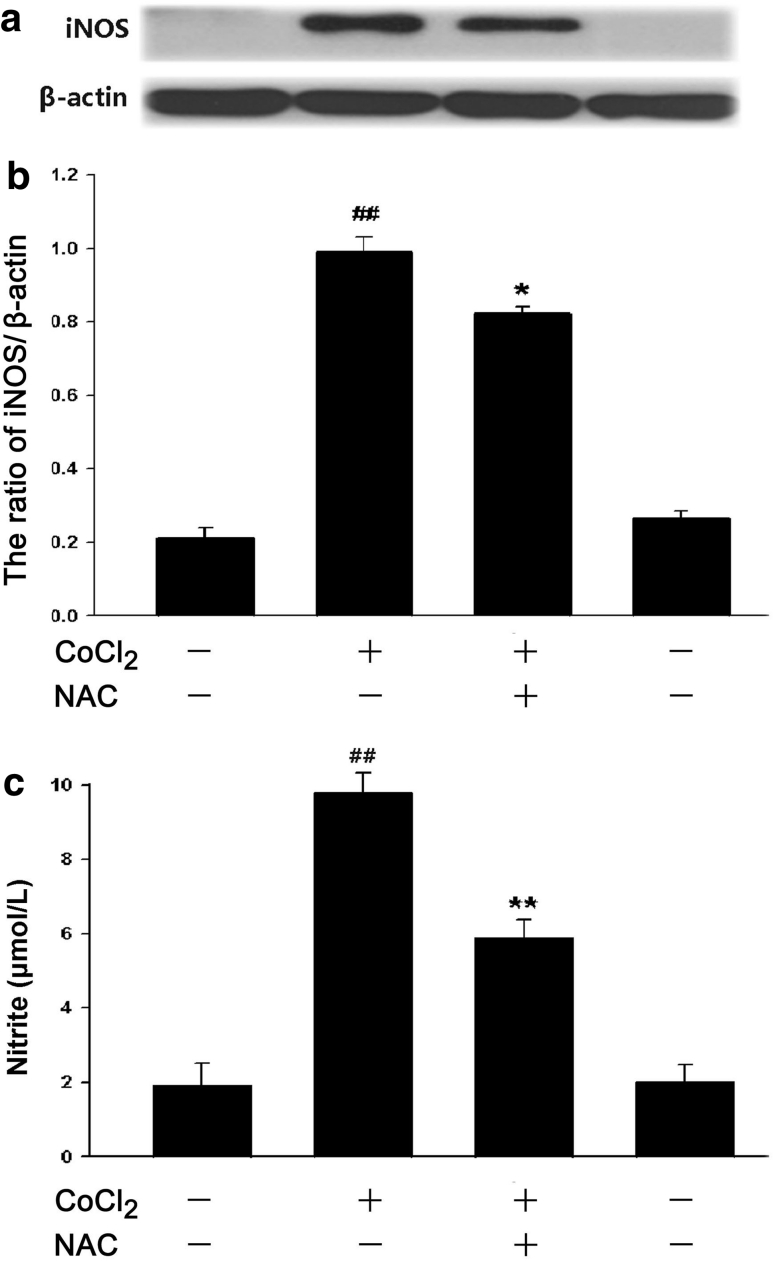
ROS contribute to CoCl_2_-induced iNOS expression and NO generation in PC12 cells. The expression of iNOS was detected by Western blot assay. **a** PC12 cells were preconditioned with 500 μmol/l NAC for 60 min before exposure to 600 μmol/l CoCl_2_ for 24 h. **b** Densitometric analysis of the results from A. **c** PC12 cells were treated with 600 μmol/l CoCl_2_ for 48 h in the presence or absence of pretreatment with 500 μmol/l NAC for 60 min. Nitrite in the culture supernatant was determined using the Griess reagent as described in “Materials and Methods”. Data were presented as the mean ± SEM (n = 5). ^##^ *P* < 0.01 versus control group; *P < 0.05, **P < 0.01 versus CoCl_2_ group

To examine whether ROS were participated in the CoCl_2_-induced expression of iNOS and production of NO in PC12 cells, PC12 cells were pretreated with 500 μmol/l NAC (a ROS scavenger) for 60 min before exposure to CoCl_2_ at 600 μmol/l for 24 h. As shown in Fig. 6a–c, treatment with CoCl_2_ induced significant increases in iNOS expression and NO production, this effect was markedly suppressed by NAC pretreatment, suggesting that the CoCl_2_-induced increases in iNOS expression and NO production are associated with ROS overproduction in PC12 cells.

### p38MAPK Activation and Oxidative Stress Mediate CoCl_2_-Induced Secretion of IL-6 in PC12 Cells

**Fig. 7:**
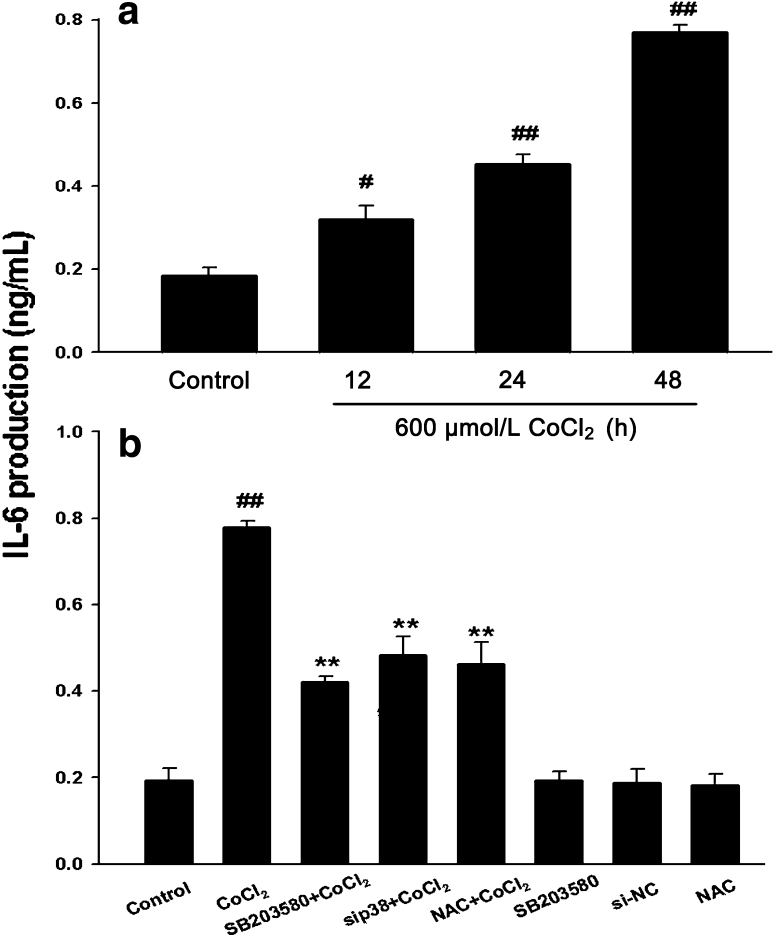
Effects of indicated treatments on secretions of IL-6 induced by CoCl_2_ from PC12 cells. **a** PC12 cells were treated with 600 μmol/l CoCl_2_ for indicated times. **b** PC12 cells were treated with 600 μmol/l CoCl_2_ for 48 h in the absence or presence of pretreatment with 20 μmol/l SB203580 for 60 min or 500 μmol/l NAC for 60 min or co-incubation with Si-p38 for 6 h. ELISA was performed to detect the levels of IL-6 in cell supernatants. Data were presented as the mean ± SEM (n = 3). ^#^ *P* < 0.05, ^##^ *P* < 0.01 versus control group; **P < 0.01 versus CoCl_2_ group

To further determine the role of activation of p38MAPK in the CoCl_2_-stimulated inflammation in PC12 cells, we examined the effect of inhibition of p38MAPK on the CoCl_2_-induced secretion of IL-6 (another important inflammatory indicator) in PC12 cells. As shown in Fig. 7a, when PC12 cells were treated with 600 μmol/l CoCl_2_ for 12, 24 and 48 h, respectively, IL-6 secretion from PC12 cells was significantly increased in a time-dependant manner. However, the CoCl_2_-induced increase in IL-6 secretion was obviously blocked by pretreatment with SB203580 (20 μmol/l) or NAC (500 μmol/l) for 60 min or co-incubation with Si-p38 for 6 h, respectively. (Fig. 7b). Alone, neither SB203580 nor NAC affected the IL-6 secretion in PC12 cells. These results suggested that p38MAPK activation and oxidative stress mediate the secretion of IL-6 induced by CoCl_2_.

### H_2_S Attenuates CoCl_2_-Induced iNOS or nNOS Expression and NO Production in PC12 Cells

**Fig. 8:**
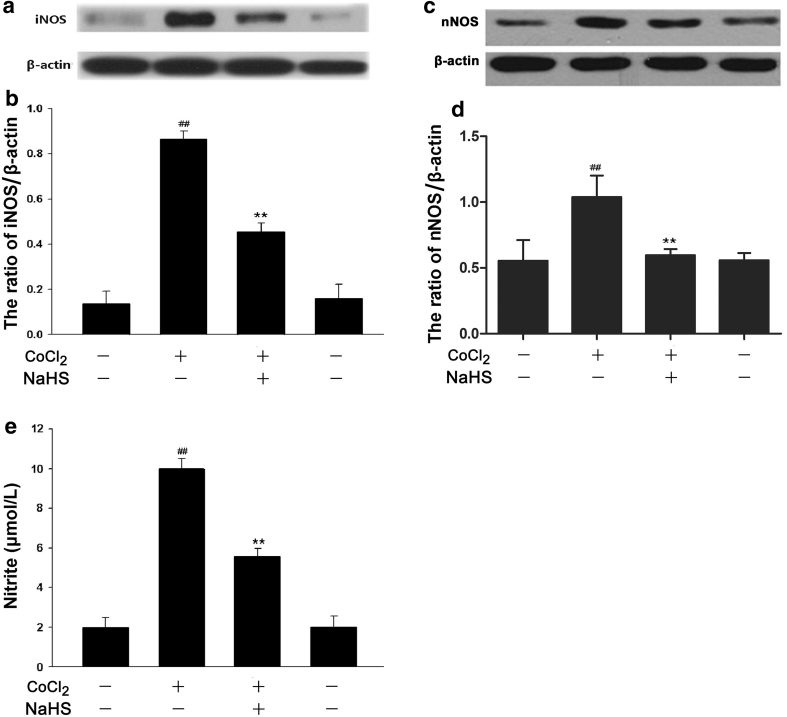
NaHS attenuates CoCl_2_-induced expression levels of iNOS and nNOS as well as NO production in PC12 cells. PC12 cells were treated with 600 μmol/l CoCl_2_ for 24 h in the absence or presence of the preconditioning with 400 μmol/l NaHS for 30 min. The expression levels of iNOS (**a**) and nNOS (**c**) were determined by Western blot assay. **b** and **d** Densitometric analysis of the results from **a** and **c**, respectively. **e** PC12 cells were treated with 600 μmol/l CoCl_2_ for 48 h in the presence or absence of pretreatment with 400 μmol/l NaHS for 30 min. Nitrite in the culture supernatant was determined using the Griess reagent as described in “Materials and Methods”. Data were presented as the mean ± SEM (n = 5). ^##^ *P* < 0.01 versus Control group; **P < 0.01 versus CoCl_2_ group

As shown in Fig. 8a and b, exposure of PC12 cells to 600 μmol/l CoCl_2_ for 24 h obviously upregulated expression of iNOS, this effect was markedly suppressed by pretreatment of cells with 400 μmol/l NaHS for 30 min before exposure to CoCl_2_. Similarly, NaHS pretreatment also attenuated the upregulation of expression of neuronal NOS (nNOS) induced by CoCl_2_ exposure (Fig. 8c, d). Additionally, treatment of PC12 cells with 600 μmol/l CoCl_2_ for 48 h enhanced production of NO, which was inhibited by pretreatment of cells with 400 μmol/l NaHS for 30 min prior to CoCl_2_ treatment (Fig. 8e). NaHS at 400 μmol/l alone did not alter the basal expressions of iNOS and nNOS as well as NO production (Fig. 8a–e). The above findings suggested that H_2_S can inhibit the CoCl_2_-induced expression of iNOS and nNOS as well as production of NO.

### H_2_S Suppresses CoCl_2_-Stimulated IL-6 Secretion from PC12 Cells

**Fig. 9:**
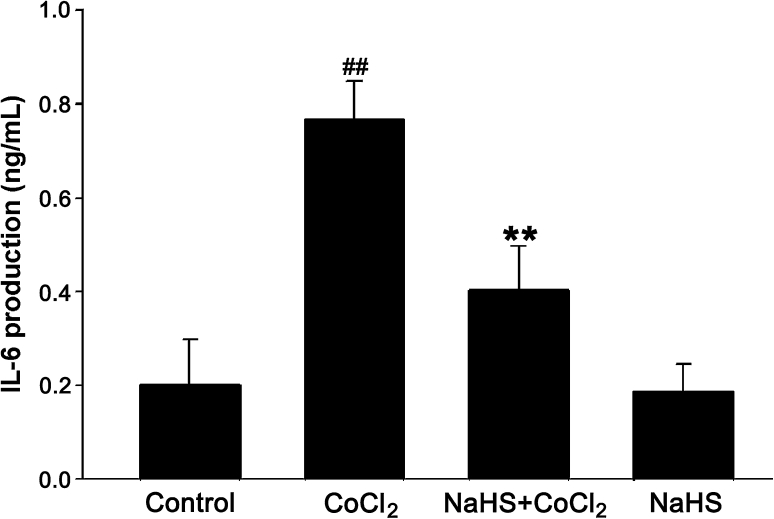
NaHS attenuates CoCl_2_-stimulated IL-6 secretion from PC12 cells. PC12 cells were treated with 600 μmol/l CoCl_2_ for 48 h in the presence or absence of pretreatment with 400 μmol/l NaHS for 30 min. ELISA was performed to detect the levels of IL-6 in cell supernatants. Data were presented as the mean ± SEM (n = 3). ^##^ *P* < 0.01 versus Control group; **P < 0.01 versus CoCl_2_ group

To further confirm the antiinflammatory effect of H_2_S, we examined the effect of H_2_S on the secretion of IL-6 by CoCl_2_. As shown in Fig. 9, after exposure of PC12 cells to 600 μmol/l CoCl_2_ for 48 h, IL-6 secretion was significantly increased, and this increase in IL-6 secretion was reduced by pretreatment with NaHS (400 μmol/l) for 30 min before exposure to 600 μmol/l CoCl_2_. NaHS at 400 μmol/l alone did not affect the basal IL-6 secretion (*P* > 0.05). Our findings suggested that H_2_S can inhibit the CoCl_2_-induced IL-6 secretion.

### H_2_S Inhibits CoCl_2_-Induced Phosphorylation of p38MAPK in PC12 Cells

**Fig. 10:**
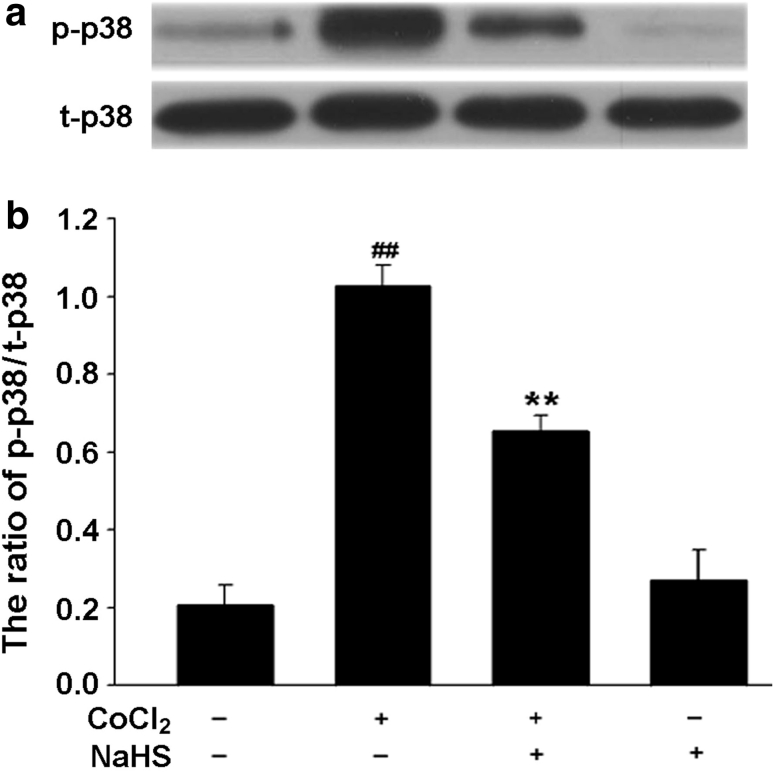
NaHS blocks CoCl_2_-induced activation of p38MAPK in PC12 cells. The expression of p-p38MAPK was determined by Western blot assay. **a** PC12 cells were treated with 600 μmol/l CoCl_2_ for 120 min in the presence or absence of pretreatment with 400 μmol/l NaHS for 30 min. **b** Densitometric analysis of the results from **a**. Data were presented as the mean ± SEM (n = 3). ^##^ *P* < 0.01 versus Control group; **P < 0.01 versus CoCl_2_ group

As shown in Fig. 10, exposure of PC12 cells to 600 μmol/l CoCl_2_ for 120 min obviously upregulated the expression of p-p38MAPK, this effect was markedly suppressed by pretreatment of cells with 400 μmol/l NaHS for 30 min before exposure to CoCl_2_. However, pretreatment with 400 μmol/l NaHS had no significant effect on the basal expression of t-p38MAPK protein (*P* > 0.05).

## Discussion

In the present study, we demonstrate for the first time, to our knowledge,that H_2_S protects against the chemical hypoxia-induced inflammation by inhibiting p38MAPK- iNOS pathway in PC12 cells. We provide novel findings to confirm the inhibitory effect of H_2_S on the neural inflammation induced by chemical hypoxia.

It is well documented that hypoxia plays a critical role in development, cellular homeostasis and pathological conditions, such as stroke. There is also increasing evidence that hypoxia is an important modulator of the inflammatory process. Thus, it is very important to explore the mechanisms responsible for hypoxia-induced inflammation or anti-neuroinflammation in different types of hypoxic model. In this study, we used the CoCl_2_-stimulated PC12 cells, which are widely used as a cellular model to investigate neuronal injury, to dissect the potential mechanisms underlying effect of inflammation on the hypoxia-induced neuronal injury and the role of H_2_S in anti-neuroinflammation.

Similar to the findings of our previous study [14] and other reports [19, 20], we found that chemical hypoxia induced significant inflammatory response, evidenced by increases in NO production and IL-6 secretion from PC12 cells. NO is a major toxic substance among several proinflammatory factors [21], and is produced by the oxidation of a terminal guanidine nitrogen of l-arginine, a reaction catalyzed by the NO synthase (NOS) [22]. To date, three isoforms of NOS have been identified: neuronal NOS (nNOS), endothelial NOS (eNOS) and inducible NOS (iNOS) [23, 24]. iNOS induces high level of NO production and is typically synthesized in response to immune/inflammatory stimuli [24]. Interestingly, we also found that CoCl_2_ considerably upregulated the expression levels of iNOS and nNOS, suggesting activation of iNOS/NO or nNOS/NO system by hypoxia stimulus. Furthermore, since previous studied have shown that CoCl_2_ can induce IL-6 secretion in several types of cells [14, 19, 20]. We further explored the effect of CoCl_2_ on IL-6 secretion in PC12 cells. Our results showed the stimulatory effect of CoCl_2_ on IL-6 secretion, suggesting that CoCl_2_ may be an inducer for IL-6 secretion.

To dissect the roles of iNOS and nNOS in the CoCl_2_-induced injuries, PC12 cells were pretreated with either L-canavanine, (an inhibitor of iNOS) or 7-NI (an inhibitor of nNOS) before exposure to CoCl_2_ for 24 h. The results of this study showed that L-canavanine and 7-NI inhibited CoCl_2_-induced cytotoxicity, indicating involvement of activation of iNOS/NO or nNOS/NO system in the CoCl_2_-induced injuries in PC12 cells. Our results is supported by the previous study showing that mice treated with NOS inhibitor or mice lacking the iNOS gene are remarkably resistant to MPTP-induced neurotoxicity [25, 26]. In nNOS knockout mice, MPTP-induced neuronal injury is depressed compared with wild type mice [25].

Numerous intracellular signal transduction pathways converge with the activation of mitogen-activated protein kinase (MAPK) family, which is composed of three main members: extracellular signal-regulated protein kinase 1/2 (ERK1/2), C-Jun-N-terminal kinase (JNK) and p38MAPK. MAPK family plays a critical role in the regulation of cell growth and differentiation and in the control of cellular responses to cytokines and stress. It was documented that p38MAPK, but not ERK1/2, is implicated in the inflammatory stimulus-induced NO production, iNOS expression and tumor necrosis factor-α (TNF-α) secretion [27, 28]. In the current study, we observed that chemical hypoxia dramatically upregulated p-p38MAPK expression, and that SB203580, a selective p38MAPK inhibitor, suppressed the CoCl_2_-induced enhancement of iNOS expression and NO production as well as IL-6 secretion. Since the pharmacological inhibitor is not specific, we further demonstrated the inhibition of p38MAPK with genetic silencing of p38MAPK by RNAi (Si-p38MAPK). Notably, genetic silencing of p38MAPK also produced the similar inhibitory effect on the CoCl_2_-induced inflammation. Combining with the above results, it was suggested that p38MAPK/iNOS pathway is involved in the chemical hypoxia-induced inflammation and injuries. Our results provide a novel evidence for the role of p38MAPK/iNOS pathway in CoCl_2_-induced inflammation and injuries. Furthermore, the results of our previous study [29] and this study revealed that p38MAPK or iNOS pathway is activated by ROS.

Importantly, we found that H_2_S plays an important role in anti-neuroinflammation in the chemical hypoxia-treated PC12 cells. Increasing evidence reveals that H_2_S may serve as a critical neuroprotective agent or neuromodulater. However, the role of H_2_S in inflammatory processes is controversial. It has been indicated that H_2_S has a proinflammatory role in several animal models [4, 5, 11, 12]. Contrarily, H_2_S has been reported to have beneficial effects in inflammatory processes. Hu et al. reported that H_2_S can attenuate the LPS-stimulated NO production and TNF-α secretion, but they did not observe the effect of exogenous H_2_S on IL-6 secretion [13]. In the human chondrocyte cell line C-28/I2, H_2_S blocks the constitutive and IL-1β-induced IL-6 and IL-8 expression [30]. In this study, we found that pretreatment with NaHS (a donor of H_2_S) obviously inhibited the chemical hypoxia-induced neuroinflammation, leading to the decreases in NO production and IL-6 secretion. Our findings are comparable with the previous studies [13, 30].

Interestingly, we found that the inhibitory effect of H_2_S on NO production is mediated by reducing the stimulatory effects of CoCl_2_ on iNOS and nNOS expression, which is comparable with the previous study reported that H_2_S inhibits not only NO production, but also iNOS expression in the LPS-stimulated microglia [13]. Additionally, our results revealed that both H_2_S and NAC (a scavenger of ROS) depressed the CoCl_2_-induced activation of iNOS/NO system and IL-6 secretion, indicating involvement of antioxidative effect in antiinflammation of H_2_S in chemical hypoxia-stimulated PC12 cells. Yang et al. also reported that H_2_S prevents the CoCl_2_-induced inflammation by reducing ROS generation in HaCaT skin cells [14], which support our findings. Furthermore, our previous study has shown that the chemical hypoxia-induced p38MAPK phosphorylation was inhibited by NAC [29], suggesting that p38MAPK pathway is activated by ROS. In this study, we also demonstrated the inhibitory effect of H_2_S on the CoCl_2_-induced activation of p38MAPK pathway, suggesting that inhibition of ROS-activated p38MAPK pathway may be another mechanism underlying the antiinflammatory effect of H_2_S. This is consistent with the previous results that MAPK signaling pathway is a target for antiinflammatory therapy [31] and that H_2_S protects microglia against the LPS-induced inflammation by inhibiting p38MAPK [13].

In conclusion, the findings of present study provide the first evidence that ROS-activated p38MAPK-iNOS pathway contributes to the chemical hypoxia-induced inflammation and injuries and that exogenous H_2_S obviously attenuates the chemical hypoxia-induced inflammatory effect, leading to decreases in NO production and IL-6 secretion from PC12 cells. The mechanisms underlying the antiinflammatory effect of H_2_S are associated with its antioxidation and inhibition of ROS-activated p38MAPK- iNOS pathway. These findings way provide new insights into therapeutic approach for prevention and treatment of the hypoxia-related neuroinflammation and injuries.
